# Epigenetic Mechanisms of Breast and Ovarian Cancer Development: Interplay Between DNA Methylation/Demethylation Enzymes, MicroRNAs, and Long Non-Coding RNAs

**DOI:** 10.3390/epigenomes10030045

**Published:** 2026-07-04

**Authors:** Svetlana S. Lukina, Irina V. Pronina, Alexander A. Bril, Alexey M. Burdennyy, Vitaly I. Loginov, Sergey G. Morozov

**Affiliations:** 1 Institute of General Pathology and Pathophysiology, 125315 Moscow, Russia; sveta_sergeevna349@mail.ru (S.S.L.); alexbril2005@gmail.com (A.A.B.); burdennyy@gmail.com (A.M.B.); loginov7w@gmail.com (V.I.L.); sergey_moroz@list.ru (S.G.M.); 2 Faculty of Natural Sciences, Federal State University of Education, 105005 Moscow, Russia

**Keywords:** DNA methyltransferases (DNMTs), ten-eleven translocation enzymes (TET), microRNAs (miRNAs), long non-coding RNAs (lncRNAs), ovarian cancer (OC), breast cancer (BC)

## Abstract

Structural and functional disruptions of the epigenome are hallmarks of breast and ovarian carcinogenesis. This review dissects the reciprocal regulatory networks co-operated by DNA methyltransferases (DNMTs), ten-eleven translocation enzymes (TETs), and key non-coding RNAs (microRNAs and lncRNAs). We map the precise molecular mechanisms through which these epigenetic modulators alter chromatin accessibility, drive transcriptional reprogramming, and promote phenotypic plasticity in hormone-dependent malignancies. By systematically contrasting the distinct yet overlapping epigenetic profiles of breast and ovarian tumors, we elucidate how these aberrations dictate clinical outcomes. This comprehensive synthesis offers critical insights into the dual utility of these epigenetic elements as dual-purpose diagnostic biomarkers and druggable therapeutic targets, laying the groundwork for next-generation targeted epigenetical therapies.

## 1. Introduction

Epithelial malignancies of the ovaries and breasts are among the most common and deadly forms of cancer among women [1]. Their heterogeneity makes them particularly important for research. Despite the development of modern diagnostic and treatment methods, survival and prognosis for metastasis in cases of delayed detection of these cancers remain significantly low, highlighting the need for in-depth studies on their molecular basis and the epigenetic factors influencing the progression of breast (BC) and ovarian cancers (OC).

The heterogeneity of female reproductive system cancers is determined by their various histopathological and/or immunohistochemical features. OC is classified into five histological types, according to the 2023 classification: high- and low-grade serous ovarian cancer, endometrioid, clear cell, and mucinous ovarian cancer. The aggressiveness of this type of cancer is associated with its proliferative and metastatic potential, particularly the process of epithelial–mesenchymal transition (EMT), during which epithelial cells adopt an invasive, migratory phenotype. It should be noted that the development of metastases in OC is considered an early event, as over 70% of cases are diagnosed in late stages. In particular, this type of tumor can metastasize to the pelvic organs as early as stage II. It is worth noting that there are three routes of OC metastasis: dissemination, lymphogenous, and hematogenous. Dissemination through the peritoneum is the most common mode of metastasis for this type of tumor. BC is more typically classified into immunohistochemical subtypes based on hormonal status and the Ki67 factor than by histopathological features. There are two luminal subtypes of BC. Luminal A is the most common hormone-dependent (ER+/PR+) well-differentiated variant of BC with a low proliferation index (low Ki-67) and the absence of HER2 overexpression. This subtype has the most favorable prognosis and is sensitive to hormonal therapy. Luminal B is detected in 20% of cases and has worse survival prognosis. It includes HER2-positive (with high protein expression) and HER2-negative (low/normal expression) variants of BC. These groups differ critically in prognosis, aggressiveness of the disease course, and need for targeted anti-HER2 therapy. There is also HER2-positive BC (10–15% of cases), which is characterized by excessive expression of the HER2/neu protein and low expression of estrogen and progesterone receptors. This is an aggressive subtype with high proliferative activity, requiring the use of targeted therapy; unlike luminal subtypes, it is hormone-independent. The most negative, from all points of view, is the triple-negative subtype of BC, characterized by the absence of expression of all of the abovementioned receptors for hormonal factors, aggressive progression dynamics, a high level of metastasis, and an unfavorable prognosis [2,3]. BC and OC have common structural changes in the BRCA1 and BRCA2 genes, as well as a number of other genes [3]. Furthermore, common patterns of epigenetic modifications that determine the dynamic regulation of processes occurring during oncogenesis have been identified for them [4].

DNA methylation and demethylation are among the key epigenetic regulators that alter gene expression [5,6]. Cytosine methylation in CpG islands is mediated by DNA methyltransferases (DNMTs) and suppresses the expression of target genes. Demethylation is associated with the activity of TET enzymes, which remove methyl groups, restoring gene expression. These processes are critical for cellular function. They are directly linked to various signaling systems involved in the cell cycle, the disruption of which contributes to tumorigenesis [5,6]. Methylation and demethylation are among the most studied epigenetic modifications in the context of carcinogenesis.

Non-coding RNAs (ncRNAs), including microRNAs (miRNAs) and long non-coding RNAs (lncRNAs), are also integral elements of epigenetic control [7]. MiRNAs are short RNAs that regulate post-transcriptional gene silencing by binding to mRNAs, suppressing expression at the translational level. lncRNAs can act at various levels, including complexing with miRNAs, modifying histones, and regulating enzymes by direct binding. NcRNAs play a critical role in numerous cellular processes, including the regulation of the cell cycle and apoptosis, and their dysfunction or overproduction often correlates with tumor development [7].

Methyltransferases, demethylases, and ncRNAs not only participate in the regulation of gene expression but also interact with each other, creating complex networks that control signaling cascades involved in cancer development. For example, miRNAs can regulate the activity of enzymes involved in DNA methylation/demethylation, while lncRNAs can bind to miRNAs, as well as proteins, and alter their activity. Studying the interactions between methylation/demethylation enzymes and ncRNAs is a highly relevant topic in modern oncology. These processes reveal key aspects of cancer pathogenesis and open new avenues for the development of therapeutic strategies. Given the significant interest in epigenetics in the context of cancer, this study contributes to our understanding of the complex epigenetic landscape of tumors and will, hopefully, serve as a foundation for future discoveries in this field.

This review focuses on epigenetic mechanisms that may underlie the malignant transformation of BC and OC. It examines key components of the development, progression, and metastasis of BC and OC, including the regulation of DNA-modifying enzymes and ncRNAs in the management of complex signaling cascades and the determination of oncogenesis and possible targeted therapy.

To assess the problem and compile this review, we used the expression ((methylation OR demethylation) AND (breast OR ovarian) AND cancer AND non-coding RNA) in the systems at https://pubmed.ncbi.nlm.nih.gov/ and https://www.google.com/?hl=ru, but only included those materials that addressed significant aspects of the target problem.

## 2. DNA Methylation and Demethylation Processes and Their Regulation in BC and OC

### 2.1. Mechanisms of DNA Methylation

**Figure 1:**
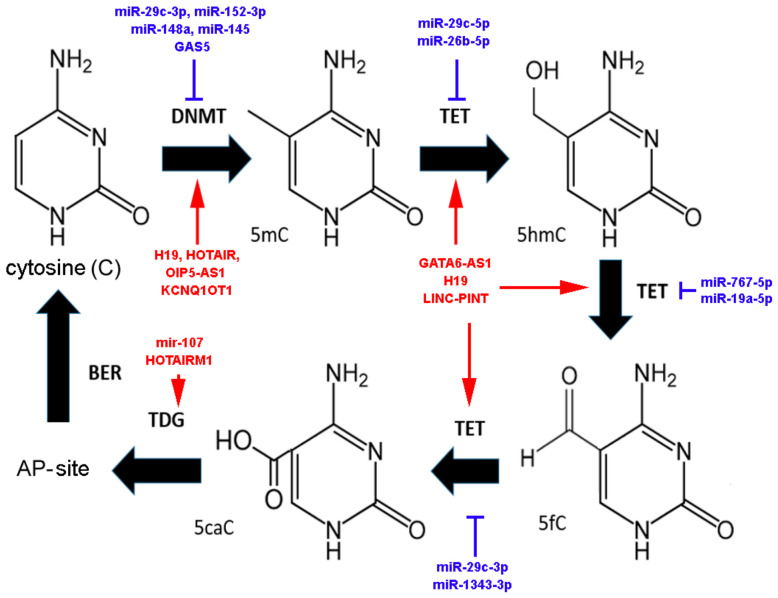
Scheme of cytosine methylation and demethylation with the enzymes involved. 5-methylcytosine (5mC), 5-hydroxymethylcytosine (5hmC), 5-formylcytosine (5fC), 5-carboxycytosine (5caC), DNA methyltransferase (DNMT), TET family DNA oxygenase (TET), specific DNA glycosylase (TDG), apurinic–apyrimidinic site (AP-site), DNA base excision repair (BER). ncRNAs that activate the process are shown in red; ncRNAs that inhibit the process are shown in blue.

One of the most significant mechanisms of epigenetic regulation, determining the potential for cell proliferation, invasion, and apoptosis, is DNA methylation, which involves the addition of a methyl group to the 5′-carbon of cytosine. This process begins with the identification of cytosine- and guanine-rich regions called “CpG islands” by the enzyme DNMT. Since CpG islands are often located in the promoter regions of genes, methylation can directly influence transcription. Tumor suppressor genes are hypermethylated in cancer, resulting in low expression. Oncogenes (proto-oncogenes) are typically demethylated, which promotes tumor progression [8,9] (Figure 1).

The methylation process occurs through the following mechanism. The Cys81 amino acid radical in the active site of the DNMT enzyme approaches the methyl donor, SAM (S-adenosylmethionine), and the substrate, cytosine (C). The elimination of a proton from the SH group of cysteine results in the formation of an active nucleophilic site at the sulfur atom. The interaction of the nucleophile with cytosine occurs as an addition at the C6 position. This reactivity is determined by conjugation within the heterocycle: the +M effect of the amino group initiates a mesomeric shift in electron density, which, combined with the polarizing effect of the double bonds, makes the C6 carbon the most favorable target for nucleophilic attack. After nucleophilic attack, the formation of a conjugated system causes the negative charge from sulfur to be uniformly distributed throughout the cyclic structure. At this stage, a third component, SAM, enters the reaction, possessing a positive charge on the sulfur atom linked to the methyl group (CH3). As an electrophile, SAM attacks the free C5 position of cytosine. This reaction demonstrates the transferase activity of the DNMT enzyme. After the transfer of the methyl group, an unstable structure is formed in which the C–H bond at the fifth carbon atom is weakened, resulting in the hydrogen atom binding to the sulfur in cysteine to form a leaving group, which is eliminated by the beta-elimination or 1,2-elimination mechanism [10]. The main end product of the reaction is 5-methylcytosine (5mC), while the by-product, S-adenosylhomocysteine (SAH), inhibits further DNMT activity through negative feedback [11].

In human cells, DNA methylation is catalyzed by three types of DNA methyltransferases (DNMTs, KEGG: EC 2.1.1.37): DNMT1, DNMT3A, and DNMT3B. The genes encoding them are located at three genomic loci on different chromosomes: DNMT1–19p13.2, DNMT3A–2p23.3, and DNMT3B–20q11.21 [12]. The DNMT1 enzyme is responsible for maintaining existing methylation patterns during replication and ensuring the invariability of epigenetic marks after several cell divisions. It acts by recognizing hemimethylated DNA, in which the CH3 group is attached to cytosine in only one strand in CpG dinucleotides, while the cytosine is unmethylated on the other strand, and adds the CH3 fragment to the newly synthesized strand. DNMT3A and DNMT3B establish the CH3 mark de novo at previously unmethylated CpG sites.

DNA methyltransferases play a central role in shaping the BC epigenome, and their deregulation is closely associated with aggressive tumor biology and poor patient prognosis. DNMT1, a key maintenance methyltransferase, is frequently overexpressed in BC, thereby ensuring the stable propagation of aberrant methylation patterns across cell generations [13]. Aberrant DNMT1 activity promotes the persistent downregulation of key tumor suppressor genes, including BRCA1, CDH1, RARβ2, GSTP1, RASSF1A, PTEN, and MSH2, leading to tumor progression and therapy resistance [14]. High DNMT1 expression correlates with high tumor grade, lymph node metastasis, and loss of estrogen/progesterone receptor expression. DNMT3B-mediated suppression of BRCA1 is associated with the disruption of chromatin domain structure, genomic instability, and transcriptional impairment, highlighting a coordinated epigenetic mechanism [15].

Data presented in The Cancer Genome Atlas (TCGA) show that DNMT expression patterns are not uniform but vary depending on the molecular subtype of BC [16]. Luminal A subtype, which is characterized by the most favorable prognosis with 10-year survival rates greater than 80%, has a lower expression of DNMT1, DNMT3A, and DNMT3B than other BC subtypes. Basal-like subtypes, luminal B, and tumors with high HER2 expression show significantly increased DNMT expression [16]. DNMT1 expression is consistently elevated in tumors across all BC subtypes compared to normal breast tissue. DNMT3A shows a similar trend: basal-like tumors show the highest expression, while HER2-enriched (ERBB2), luminal A, and luminal B subtypes show moderate but elevated levels compared to normal tissue. DNMT3B expression is particularly pronounced in basal-like and HER2-enriched tumors, while luminal A and B tumors show comparatively lower expression, closer to that in normal tissue. Taken together, these data suggest that high levels of DNMT1, DNMT3A, and DNMT3B expression contribute to BC pathogenesis, with basal-like and HER2-enriched tumors showing the greatest dependence on DNMT-driven epigenetic regulation, highlighting the potential of these enzymes as predictive biomarkers and therapeutic targets [16,17].

The results of studies on DNMT expression in OC are controversial, which is likely due to differences in the histological subtypes of studied tumors, patient groups, and the methods used [18,19]. Based on a comprehensive analysis of DNMT expression throughout the progression of OC, the authors of [18] showed that DNMT1 and DNMT3A appear to play a major role in tumor initiation, while DNMT3B plays a major role in tumor progression. The expression level of DNMT1 was increased in recurrent tumors compared to primary tumors, and the mRNA levels of DNMT3A, DNMT1, and DNMT3B correlated with overall survival. Other researchers have hypothesized a model of carcinogenesis in which DNMT3A and DNMT3B are specifically recruited during tumor initiation and development with subsequent downregulation of their expression, while DNMT1 is involved in tumor progression [20]. These data support the hypothesis that alterations in DNMT expression may contribute to the development and progression of high-grade ovarian carcinomas.

In modern cancer therapy, genes of the DNA methyltransferase family are of particular interest as targets for inhibitors that reduce their expression in cancer cells. In cell models of BC and OC, active restoration of a number of methylation-inactivated suppressor genes has been demonstrated following cell treatment with demethylating agents [21,22].

### 2.2. The Mechanism of DNA Demethylation and Its Role in the Development of BC and OC

An antagonist of DNA methylation is demethylation, which is also a key process involved in regulating gene expression without altering the nucleotide sequence. DNA demethylation is a multistep biological process that involves the removal of methyl groups from the C5 position of cytosine within CpG islands (Figure 1). This process regulates gene expression by restoring DNA accessibility to transcription factors and polymerases and occurs in two main forms: active and passive. Passive DNA demethylation is caused by a decrease in the activity or absence of DNMTs, while active demethylation is carried out by enzymes oxidizing the TET (Ten-Eleven Translocation, KEGG: EC 1.14.11.80) family [23], which include TET1, TET2, and TET3. These enzymes have a catalytic domain in the C-terminal region and perform similar functions. The CXXC domain, located at the N-terminus of Tet1 and Tet3, but not Tet2, gives the enzymes the ability to directly bind DNA in the region of CpG islands, which allows DNA to be protected from aberrant methylation. TET1 is located on chromosome 10 at locus 10q21.3 and contains 19 exons, TET2 at 4q24 (14 exons), and TET3 at 2p13.1 (17 exons). One Tet1 isoform lacks the CXXC domain, which reduces DNA binding efficiency and confirms the functionality of this fragment. IDAX (also known as CXXC4 or CXXC finger protein 4) is believed to play a role in regulating TET2 activity by facilitating its recruitment to CpG regions. Thus, the level and cellular localization of IDAX may play a role in targeting Tet2 to specific sites and determining its activity [24,25].

Another enzyme involved in demethylation processes is TDG (KEGG: EC 3.2.2.29), which belongs to the class of DNA glycosylases that repair DNA by hydrolyzing the carbon–nitrogen bond between the sugar–phosphate backbone of DNA and the erroneous base. This enzyme plays a central role in cellular defense against genetic mutations caused by the spontaneous deamination of 5mC and cytosine, as well as other modifications of nitrogenous bases.

During the demethylation process, TET oxygenases use α-ketoglutarate (α-KG), iron (Fe(II)), and oxygen (O_2_) as cofactors and catalyze the oxidation of 5-methylcytosine (5mC) to 5-hydroxymethylcytosine (5hmC), 5-formylcytosine (5fC), or 5-carboxycytosine (5caC) depending on the stage of demethylation (Figure 1). A specific DNA glycosylase (TDG, thymine DNA glycosylase) recognizes 5fC and 5caC and removes them from DNA, initiating an active base excision repair (BER) pathway, which results in the replacement of the modified cytosine with an unmethylated one through the formation of an AP site (apurinic–apyrimidinic site), into which cytosine is subsequently incorporated during BER mediation by the uracil DNA glycosylase family, such as UNG (uracil DNA glycosylase), TDG or SMUG1 (single-strand-selective monofunctional uracil-DNA glycosylase 1), and MBD4 (methyl-CpG binding domain 4, DNA glycosylase) [26,27].

Over 10 years ago, it was reported that all three members of the TET gene family, as well as TDG, have low expression levels in various types of tumor tissues, including BC and OC, compared to adjacent normal tissues [28,29,30]. Low expression of TETs resulted in increased EMT in BC cells, leading to tumor invasion and metastasis [31]. Nevertheless, in [32], it was shown that high TET1 levels were associated with low levels of immune response markers in triple-negative BC. Reduced TET1 expression levels correlated with advanced stages in patients with high grade serous OC [33]. Interestingly, TET1 expression levels consistently increase from normal fallopian tube epithelium to invasive high grade serous OC [34]. TET1 promotes cell migration, proliferation, and chemoresistance, especially resistance to cisplatin and taxol, and tumor growth in vivo through the re-expression of Yamanaka factors and cancer stem cell marker genes (ALDH1, NANOG, OCT4, SOX2, c-Myc) in A2780, HeyC2, and SKOV3 OC cells. Moreover, TET1 increases the expression of genes involved in oncogenic signaling pathways, such as RAS/RAF, ERBB2, VEGF, TGF-β, and EGFR, and induces the expression of the CK2α gene by increasing the level of 5hmC in its promoter, which enhances tumor growth in vivo [34].

In [35], the authors suggest a tumor-suppressive role for the TET2 in hormone-dependent BC. Depletion of TET2 leads to selective DNA methylation of enhancers and subsequent disruption of transcription factor binding and transcription inactivation of a number of genes, including programmed cell death genes. TET2 can also inhibit the migration and invasion of BC cells through the demethylation of the promoter region of the E-cadherin gene and its activation [36]. Low TET2 expression correlates with late stages, a high degree of differentiation, metastatic lymph node involvement, vascular thrombosis, poor overall survival, and poor progression-free survival in patients with epithelial OC, especially the endometrioid subtype [37,38].

TET3 expression levels and 5hmC are associated with tumor hypoxia, high differentiation grade, and poor overall survival [39]. High TET3 expression correlates with better progression-free survival in BC patients treated with anthracycline chemotherapy [40]. TET3 expression is higher in borderline OC and in highly differentiated tumor cells and decreases depending on the clinical stage and tumor differentiation grade, correlating with poor overall survival [41,42].

Low 5hmC levels correlate with advanced stages, high grade differentiation, lymph node metastasis, vascular thrombosis, and poor overall survival in patients with OC [38]. 5hmC levels are decreased in cisplatin-resistant patients compared with cisplatin-responsive patients, and low 5hmC levels correlate with poor overall survival and progression-free survival after cisplatin-based chemotherapy [43].

Thus, DNMT/TET enzymes play a key role in DNA methylation/demethylation (passive or active), and their dysregulation can affect both protein-coding genes associated with cancer, such as migration, invasion, and apoptosis genes, and non-coding RNA genes. Moreover, a comparative analysis of the methylation landscapes of BC and OC has revealed both common features, likely related to the shared molecular changes occurring in these cancer types, and specific ones. For example, a significant number of studies consider the aberrant methylation of genes involved in the mechanisms of maintaining genomic integrity—homologous recombination and base excision repair, specifically the BRCA1/2 genes—to be a significant event. This process is reported to be associated with the development of triple-negative BC and high-grade serous OC. In particular, methylation of the BRCA1 gene serves as a prognostic marker of the response to PARP inhibitors and platinum-based chemotherapy [9,44]. Moreover, according to the results of international studies, biomarkers specific to each type of cancer are also identified. Thus, for BC, in addition to the BRCA1/2 genes, hypermethylation is most frequently detected for three protein-coding genes: RARβ2, GSTP1, and RASSF1A. A significantly higher level of their methylation in the tumor has been shown compared to normal tissue. In BC, the hypermethylation of genes involved in DNA repair processes, xenobiotic detoxification, hormonal and receptor signaling, and cell cycle control has been shown [14,45,46,47]. It should be noted that hypermethylation of the BRCA1 and RASSF1A genes is also detected in OC [46]. However, hypermethylation of SIM1 and ZNF154 is specific for OC [47]. Methylated genes are selected based on the results of whole-genome sequencing in samples with a cancer cell fraction of at least 20% [48]. Moreover, in our earlier studies, we demonstrated a relationship between high methylation levels of a number of protein-coding genes (e.g., RASSF1A, RARB2, SEMA3B), microRNA genes (e.g., MIR129-2), and lncRNAs (e.g., MEG3, ZNF667-AS1, SEMA3B-AS1, and SNHG6) and a significant decrease in their expression levels in BC and OC, which is apparently associated with impaired methylation/demethylation processes in tumor cells [49,50,51].

## 3. Non-Coding RNAs and Their Role in Carcinogenesis

In epigenetic regulation, key mechanisms shift from DNA methylation/demethylation and changes in chromatin structure (e.g., methylation of individual amino acid residues) to the post-transcriptional action of ncRNAs on protein synthesis. Chromatin structure, controlled by methyl groups, determines which ncRNAs are synthesized. NcRNAs, in turn, can recruit TET enzymes for demethylation or DNMTs for methylation, creating a feedback loop. Thus, DNA methylation/demethylation and ncRNAs form a unified system, where ncRNAs can act as “users” of methylation information or “instructors” for enzymes that alter DNA methylation status.

NcRNAs are transcripts from non-coding regions of the genome that regulate the expression of genes involved in various processes of cellular homeostasis. Depending on their length, they are divided into short (small) and long non-coding RNAs. Small RNAs include molecules shorter than 200 nucleotides: miRNAs, piwiRNAs, small interfering RNAs, etc. LncRNAs include molecules longer than 200 nucleotides that have a linear or circular structure (circular RNAs), and they have a defective reading frame and are incapable of encoding a protein structure; however, some have been shown to encode short peptides. The division by length is arbitrary. MiRNAs and lncRNAs have the subjects of several studies, and are therefore promising as biomarkers. As with protein-coding genes, ncRNA genes are also characterized by methylation, which, under certain conditions, can become a factor leading to the development of malignant transformation [52].

### 3.1. Biogenesis of MicroRNAs and Their Oncogenic and Tumor-Suppressive Roles

MiRNAs are small non-coding RNA molecules approximately 22 nucleotides long that play a key role in the post-transcriptional regulation of gene expression. They function by binding to target mRNAs, leading to the inhibition of their expression or complete degradation. In cancer, miRNAs act as important regulators of the cell cycle, contributing to both tumor development and progression and to their suppression [53].

**Figure 2:**
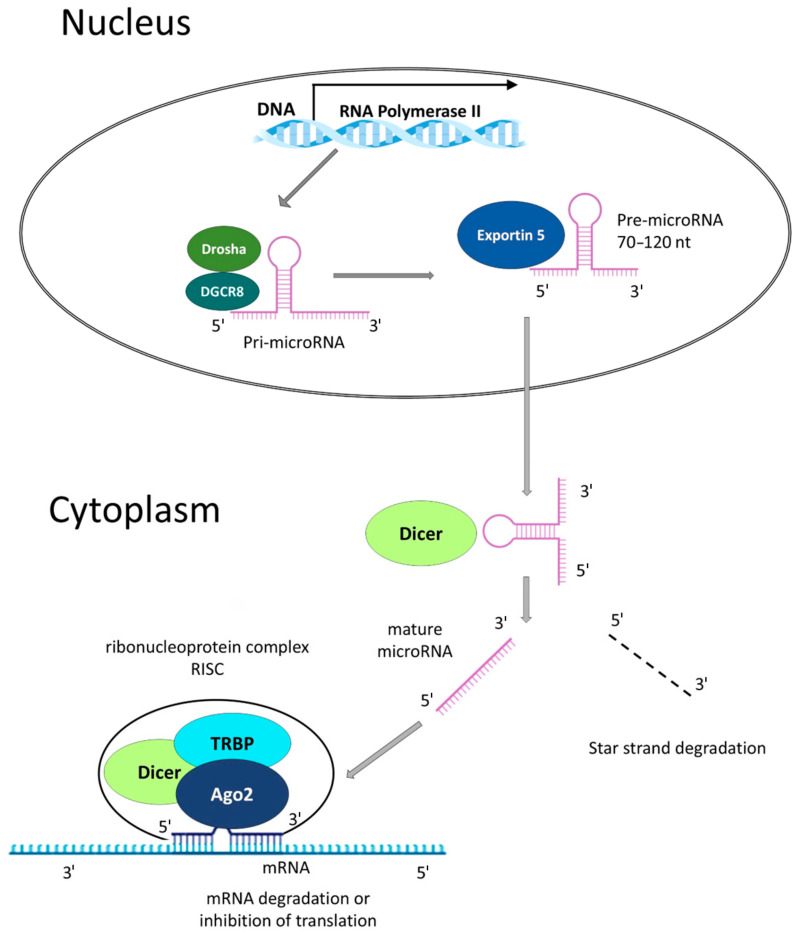
Mechanism of microRNA expression, processing, and biogenesis. RNA polymerase II; RNase III (Dicer; DICER1); RISC (RNA-induced silencing complex), TRBP (Trans-Activation-Responsive RNA-Binding Protein), Ago2 (Argonaute 2, RISC Catalytic Component). Gray arrows indicate the direction of miRNA processing and transport. Pink structures represent pri-miRNA, pre-miRNA and mature miRNA molecules. Different colors are used only to distinguish proteins and molecular components.

The biogenesis of miRNA consists of three main stages. First, a fragment located in the intragenic region or intergenic space is transcribed by RNA polymerase II (in some cases, synthesis by RNA polymerase III is possible) (Figure 2), which produces RNA approximately 3000–5000 bases long, called primary microRNA (pri-miRNA), and forms specific hairpin structures recognized by the Drosha-DGCR8 complex, often referred to as the “microprocessor”. In the second stage, pri-miRNA is shortened by the DROSHA and DGCR8 enzymes, forming a 70-nucleotide structure called the precursor microRNA (pre-miRNA) [54,55]. A non-canonical mechanism of pre-miRNA formation through alternative splicing of intron regions, called “mirtrons”, is also possible [56]. In the final step, the export receptor exportin-5 (XPO5) directly interacts with the pre-miRNA, transporting it into the cytoplasm, after which Dicer cleaves the pre-miRNA into short fragments of 22 bases [57,58]. Its double-stranded structure then dissociates into a single-stranded structure. AGO-2 binds to one of the mature strands, forming the RNA-induced silencing complex (RISC) [59]. The second strand, called the star strand (‘*’), is usually degraded (Figure 2).

Depending on the functions of their target mRNAs, miRNAs can act as oncogenes or tumor suppressors. In OC, a decrease in the expression level of tumor suppressor miRNAs miR-101, miR-193b, miR-27b-5p, miR-125a, and miR-125b was observed, along with an increase in the expression level of oncogenic miRNAs miR-4732-5p, miR-141, miR-200a, miR-7, and miR-203a [60]. The level of miR-125b decreases in BC, promoting cell proliferation and cell cycle progression [61,62]. There are numerous examples of interactions between miRNAs and mRNAs of cell cycle regulators—p53, RREB1 (Ras-Responsive Element-Binding Protein 1), c-Myc, and PTEN. For example, the transcription factor c-Myc promotes increased expression of miR-17/92, miR-21, and miR-10b, which accelerate tumor cell growth and proliferation. At the same time, c-Myc inhibits the synthesis of miR-34, let-7, and miR-15a/16-1, which perform a suppressor function [63].

Studies on molecular landscape changes in cancer have noted a link between methylation caused by the expression of DNMT family genes and a number of miRNAs with suppressor potential. At the same time, DNMT transcripts themselves can be subject to RNA interference by miRNAs, which leads to the suppression of their expression and a decrease in the protein level in the cell [64]. Thus, DNMT3A expression was significantly reduced in the presence of miR-29c-3p, and in BC, it was found that miR-152 can bind to the DNMT1 mRNA, thereby reducing the protein level (Figure 1) [62,65].

Most frequently, miRNAs suppressed by the methylation of their genes are involved in the proliferation, migration, invasion, and stemness processes of cells. Thus, it has been shown that with the hypermethylation of the MIR203 promoter region, the level of miR-203 expression is sharply reduced, which leads to overexpression of the mesenchymal marker SNAIL2. This event leads to EMT initiation, increased proliferation, invasion, an increase in the stem properties of BC cells, and metastasis formation. Functional tests to enhance the expression of miR-203 performed on the MDA-MB-231 cell line corresponding to the triple-negative phenotype of BC have shown the opposite effect reducing the tumor activity of the cells [62,66].

Hypermethylation of the MIR375 promoter region significantly reduced the expression level of miR-375 in OC. Moreover, in model experiments on OC cell lines, demethylation of MIR375 restored the normal expression level of miR-375 and led to a significant suppression of invasion, migration, and cell viability due to the activation of apoptosis. Reduced expression of miR-375 in tumor cells contributed to an increase in the expression level of YAP1, which led to hyperexpression of Wnt1 and β-catenin proteins and the initiation of the Wnt/β-catenin signaling pathway. This and other miRNA-mRNA axes may underlie the development and progression of OC [67].

### 3.2. Long Non-Coding RNAs in BC and OC

Another class of regulators involved in complex interactions during the development of BC, OC, and other cancers are lncRNAs. These represent a class of RNAs whose cellular functions are being actively studied but remain largely unknown. Despite their name, some lncRNAs encode peptides that may play a significant role in the pathogenesis of cancer and other diseases [68].

Like mRNAs, lncRNAs are transcribed by RNA polymerase II (some also by RNA polymerases I and III), spliced, subject to 5′-capping, and polyadenylation at the 3′-end, although the nature of these modifications may vary depending on the RNA type and sometimes classical modifications may be absent [69,70]. Most frequently, after processing, lncRNA acts as ceRNA (competitive endogenous RNA), affecting other types of RNA (mainly mRNA or miRNA). Interaction with miRNA can restore the expression of its target mRNA according to the “suppression of inhibitor” principle by disrupting the functioning of the RISC complex. lncRNAs are also capable of interacting directly with proteins, changing the conformation of the bound molecule, which can lead to the disruption of enzyme function [71]. In addition to regulation at the post-transcriptional and post-translational levels, lncRNAs can regulate gene expression at the epigenetic level by binding to the repressor proteins of the Polycomb system (PRC1 and PRC2) and facilitating these complexes’ binding to the target gene. Well-known examples include the lncRNAs XIST and HOTAIR, which bind to specific subunits of repressor proteins and inhibit loci associated with the X chromosome and HOX genes, respectively [72]. Many lncRNAs target general transcription factors or RNA polymerase II itself, thereby influencing the transcription of gene clusters at the basal level. For example, under stress conditions for the cell, lncRNA B2, which is a non-coding transcript of RNA polymerase III (small non-coding RNA polymerase III transcript, B2 RNA, ~180 bp long), regulates global adaptation mechanisms by inhibiting RNA polymerase II to reduce transcription at some loci [73].

There are two main principles for classifying lncRNAs: by mechanism of action and by location in the genome [74]. By mechanism of action, lncRNAs can be divided into “signaling” lncRNAs, which, under the influence of a specific external stimulus, act on proteins that regulate gene expression; “bait” lncRNAs, which repress gene transcription, interacting, for example, with transcription factors; “guide” lncRNAs for proteins transported to specific loci in DNA; and “complex” lncRNAs (a subtype of guide lncRNAs), which induce the formation of a protein complex (as in the case of HOTAIR and Polycomb). By location in the genome, lncRNAs are divided into intergenic (lincRNAs), antisense, sense, and divergent (or bidirectional). LincRNAs are the largest group of lncRNAs (about 50% of all detected ones), which are located at a distance of about 1000 bp from protein-coding genes. The functions of intergenic RNAs are more difficult to determine than for other types, as there is no one-to-one correspondence to a specific sequence, requiring the use of various chromatin mapping methods to determine effects on individual loci. This type of lncRNAs can act as an enhancer or silencer for a specific gene located significantly distant from the intergenic RNA coding sequence. Antisense lncRNAs (representing 35–40% of all lncRNAs) overlap with protein-coding genes and are transcribed from a DNA strand complementary to the coding strand. They are divided into two classes: intronic (if the transcript is completely within the boundaries of the opposite intron) and natural antisense transcripts (NATs), which partially overlap in the promoter or terminator region of the coding gene. The third class includes sense lncRNAs, which can also be divided into intronic and overlapping. Such transcripts are located on the same strand and are transcribed in the same direction as the protein-coding gene. The fourth and rarest class is bidirectional/divergent lncRNAs located on the antisense strand. Their peculiarity is the location of the transcription start point close to that of protein-coding genes, but transcription occurs in the opposite direction [74,75]. Given the complexity of detecting, classifying, and determining the functions of such a heterogeneous group of RNAs, lncRNAs from similar mRNA sequences are separated using mathematical modeling and neural networks based on a library of k-dimensional regions, which is one of the most accurate and promising methods for predicting transcript properties [76].

In BC and OC, lncRNAs are involved in complex interactions that determine further pathways of tumor progression. lncRNAs such as H19, MEG3, NEAT1, GAS5, HOTAIR, PCAT1, and KCNQ1OT1 are expressed in virtually all tumor cells; MALAT1, H19, MEG3, HOTAIR, and PVT1 play a role in disease progression [77]. In OC, deviations in the activity of dozens of lncRNAs involved in the processes of metastasis, proliferation, apoptosis, and resistance to chemotherapy were identified. For example, lncRNAs TP73-AS1, CCAT1, MALAT1, TUG1, ANRIL, LINK-A, HOTAIR, SNHG1, and SNHG16 are oncogenic, while GAS5, MEG3, and NBAT1 are, on the contrary, suppressive [78]. In BC, it has been shown that HOTAIR, MALAT1, BCAR4, H19, NEAT1, CCAT1, DANCR, MEG3, and XIST can provoke tumor formation, and GAS5, NKILA, TUG1, and PTENP1 contribute to the suppression of cancer-related cascades [79]. Just like microRNA genes and protein-coding genes, lncRNA genes undergo CpG methylation of the promoter region, which negatively affects the level of their expression [80,81].

In investigating possible pathways for the development and progression of OC, an interaction axis was established between NEAT1 and miR-214-3p, which negatively regulate each other. Moreover, overexpression of NEAT1 enhanced tumor cell proliferation, migration, and invasion, significantly reducing apoptotic activity. Overexpression of NEAT1 was associated with increased expression of the SEMA4D target gene, which is associated with angiogenesis [82]. Increased expression of the lncRNA HOTAIR, which acts as a sponge for miR-214-3p and miR-217 microRNAs, in OC leads to increased expression of PIK3R3 and triggers the PI3K/Akt/mTOR signaling cascade, which provokes OC progression [83].

LncRNAs can directly interact with genes of the DNA methyltransferase family, determining the subsequent methylation profile. LINC00518, through the DNA methyltransferase family, promotes hypermethylation of the CDX2 gene, which leads to the activation of the Wnt/β-catenin signaling pathway and the subsequent development and progression of BC, including metastasis. Demethylation of the BECN1 gene encoding Beclin1, mediated by the H19/SAHH/DNMT3B axis, contributed to the development of tamoxifen resistance in BC [80].

## 4. Interaction of LncRNA–miRNA–mRNA and Methylation/Demethylation Processes in Key Signaling Pathways of BC and OC: Relationship with Anticancer Therapy

BC and OC are characterized by a variety of genetic and epigenetic aberrations that play a key role in their development and progression. Despite the high significance of mutations influencing the onset and course of oncological diseases, pathological processes are underpinned by factors involved at various levels: genomic (different types of mutations and chromosome aberrations), epigenomic DNA modifications (modification of nucleotides by DNMT, TET, and TDG enzymes), post-transcriptional (RNA interference via miRNA and lncRNA), and post-translational (binding of lncRNA to DNMT enzymes). An imbalance between the activity of methylases and demethylases disrupts normal cellular function and promotes cancer progression [40]. An imbalance in the lncRNA–miRNA–mRNA system, maintained by aberrant methylation, is a driving force behind carcinogenesis, making these interactions a subject of active study, including as a promising target for therapy. Here, we analyze empirical examples illustrating both the ncRNA-mediated modulation of gene methylation and the reciprocal impact of DNA methylation on ncRNA expression.

In a study on the role of dioxygenases in various interactions that contribute to cancer development, it was shown that TET2 bound with lymphoid-specific helicase (LSH), which played a significant role in maintaining genomic stability by balancing 5-hmC levels in pericentromeric satellite repeats of heterochromatin. Concurrently, LSH increased TET2 expression by suppressing miR-26b-5p and miR-29c-5p, while its expression level was quite stable and resistant to DNA damage induced by cisplatin. The authors’ data [84] indicate that 5-hmC can serve as a marker of cancer metastasis and that decreased LSH expression is likely a mechanism of genomic instability underlying the loss of 5-hmC in cancer.

Aberrant patterns of miRNA expression correlate with the Warburg effect (aerobic glycolysis) in cancer cells. In [85], the authors showed that miR-145 negatively correlated with DNMT3A expression at the cellular/tissue levels and inhibited the Warburg effect by targeting HK2 mRNA. miR-145-mediated downregulation of DNMT3A expression occurred through direct action on the 3′-untranslated regions (3′-UTR) of its mRNA. However, DNMT3A knockdown was shown to increase miR-145 expression and decrease methylation in the promoter region of the miR-145 precursor gene, indicating an important interaction between miR-145 and DNMT3A through double negative feedback. The feedback loop between miR-145 and DNMT3A is a powerful indicator of the Warburg effect in OC, which promises to be a potential target for improving anticancer therapy [85].

LncRNAs play a significant role in cancer progression, being involved in numerous signaling cascades, such as PI3K/AKT, Wnt/β-catenin, MAPK, and others. The main regulators of lncRNA activity in these processes are the RNA-binding proteins STAT3, ELAVL1, and EWSR1, as well as the EZH2 protein (enhancer of zeste 2 polycomb repressive complex 2 subunit) and various miRNAs, the interactions with which competing endogenous RNAs (ceRNAs) appear to be the most significant. Thus, it was reported that the antisense lncRNA GATA6-AS1 is involved in the progression of OC. Overexpression of GATA6-AS1 significantly suppressed the proliferation, migration, and invasive capacity of OC cells, while a decrease in GATA6-AS1 levels had the opposite effect. Moreover, GATA6-AS1 bound miR-19a-5p, suppressing its expression, and indirectly increased TET2 expression. Taken together, the results of this study suggest that GATA6-AS1 may suppress the proliferation, migration, and invasive ability of OC cells by regulating the miR-19a-5p/TET2 axis [86].

**Figure 3:**
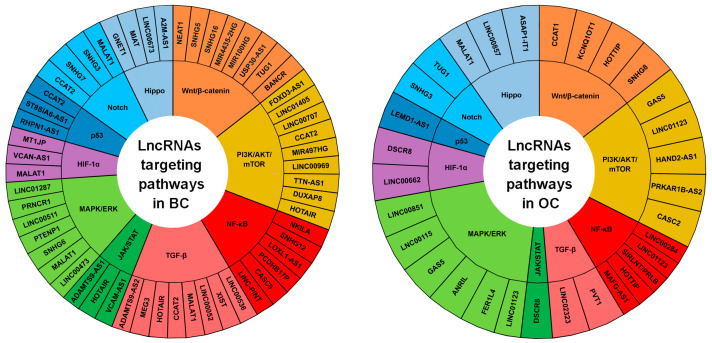
Major signaling cascades and lncRNAs targeting them in OC and BC.

Commonly, in the pathogenesis of OC and BC, aberrant signaling cascades that affect cell proliferation, angiogenesis, and metastasis are involved [87,88]. Cancer progression is closely associated with the activation of multiple signaling pathways, the most common of which are PI3K/AKT, Jak/STAT, Wnt/β-catenin, MAPK, NF-κB, Nrf2, HIF-1α, Notch, Hedgehog, and TGF-β [89], in which all of the abovementioned elements of epigenetic regulation participate (Figure 3).

### 4.1. The PI3K/Akt1/mTOR Signaling Cascade

The PI3K/Akt1/mTOR signaling cascade is associated with signal transduction from receptor tyrosine kinases (RTKs) to PI3K (phosphatidylinositol 3-kinase). Activated PI3K phosphorylates the phospholipid PIP2 (phosphatidylinositol-4,5-bisphosphate) on the cell membrane, converting it to PIP3 (phosphatidylinositol-3,4,5-trisphosphate). This leads to the phosphorylation of AKT1 (AKT serine/threonine kinase 1) via PIP3. AKT1, in turn, induces the activity of the mTORc1 complex (through the “suppression of inhibitor” TSC1,2 (TSC complex subunit 1/2) mechanism), which is responsible for regulating protein metabolism and cell growth. In turn, the mTORc2 complex activates the AKT1 enzyme, closing the regulatory loop. In addition to this axis, AKT1 is involved in the inhibition mechanism of tumor suppressors p21 and p27, as well as other factors that promote apoptosis. In the case of OC and BC, disturbances often occur due to the inhibition of the PTEN (phosphatase and tensin homolog), for example, through aberrant methylation of its promoter regions, which is possibly associated with the abnormal expression of a number of microRNAs. Thus, it was shown that overexpression of DNMT1 is associated with hypermethylation of the CpG islands of the MIR152 gene and inactivation of miR-152-3p in BC and OC [90]. The effect of miR-152-3p on the DNMT target mRNA leads to a decrease in the hypermethylation of tumor suppressor genes (e.g., PTEN, RASSF1A) and restores their expression. miR-152-3p inhibited the PI3K/AKT/mTOR pathway by downregulating PIK3CA by directly targeting its 3′-UTR in MCF-7 and MDA-MB-231 BC cell lines [90]. PI3K/protein kinase B (Akt) phosphorylation was enhanced by FOXD3-AS1 lncRNA but attenuated by miR-363. PI3K/Akt inhibition blocked FOXD3-AS1 activity and reduced tamoxifen resistance in T47D and MCF-7 BC cell lines. Taken together, this study showed that FOXD3-AS1 binds miR-363 while upregulating TFF1 (trefoil factor 1) expression, which leads to the activation of the PI3K/Akt signaling pathway and antiestrogen resistance in BC cells [91]. The increased expression of mTOR induced by miR-29b and miR-497 may also result from AKT overexpression and phosphorylation [92]. The increased expression of CCND1 and CCND2 target genes, along with AKT1, AKT3, and mTOR, can trigger cell proliferation [93]. Meanwhile, overexpression of lncRNA LINC01405 significantly decreased the expression of miR-29b and miR-497, which resulted in the inactivation of the PI3K/Akt/mTOR signaling pathway.

Further studies have shown that high expression of lncRNA LINC00707, which competitively binds to miR-423-5p, increase the expression of MARCH2, which ultimately promotes the progression of triple-negative BC and autophagy through the activation of the PI3K/AKT/mTOR signaling pathway [94]. Nonetheless, activation of the PI3K/AKT signaling pathway led to the suppression of LINC0096, which resulted in a decrease in the proliferation, migration, invasion, and metastasis of BC caused by the interaction along the LINC00969/miR-425-5p/HOXD8/ILP2 axis [95]. The cell cycle and apoptosis regulator lncRNA CCAT2, which functions through the miR-145-5p/AKT3/mTOR axis, is also involved in this signaling pathway. Moreover, CCAT2 is considered a therapeutic target for increasing the sensitivity of cells resistant to tamoxifen [96].

Tamoxifen is widely used to treat patients with estrogen receptor-positive (ER+) BC, and acquired resistance to tamoxifen is a serious problem in BC therapy. The results of several studies show that lncRNA MIR497HG deficiency induces BC progression and resistance to tamoxifen due to decreased expression of miR-497/195. MicroRNAs miR-497 and miR-195 coordinately suppress five positive regulators of the PI3K/AKT signaling pathway (MAP2K1, AKT3, BCL2, RAF1, and CCND1), leading to the inhibition of this signaling pathway. Inactivation of the PI3K/AKT pathway in tumor cells resistant to tamoxifen restores sensitivity to this drug [97]. It has been shown that the ZEB1 protein, interacting with HDAC1/2 and DNMT3B proteins provokes histone deacetylation and hypermethylation of the MIR497HG promoter, which contributes to the progression of BC and resistance to tamoxifen through the PI3K/AKT signaling pathway [97]. Thus, MIR497HG can be used as a biomarker for predicting sensitivity to tamoxifen in patients with ER+ BC. The authors of [98] showed that lncRNA TTN-AS1 can contribute to tamoxifen resistance in BC by modulating the miR-107/ZNRF2 axis and stimulating the PI3K/AKT signaling pathway. lncRNA DUXAP8 suppressed PTEN expression and increased the phosphorylation of components of the PI3K/Akt/mTOR signaling pathway. DUXAP8 also increased the expression of EZH2, which suppressed the expression of E-cadherin and RHOB, further promoting tamoxifen resistance development. DUXAP8 inactivation restored the sensitivity of BC cells to radiation therapy. Treatment with PI3K inhibitors reduced the viability of cells overexpressing DUXAP8, thus confirming its role as a mediator of tamoxifen resistance [99].

Nimbolide, a natural triterpenoid present in the edible parts of the neem tree (Azadirachta indica), appears to be a promising therapeutic agent for BC treatment. It inhibits the expression of aldose reductase (AR), subsequently blocking the IGF-1/PI3K/Akt and HIF-1α/VEGF signaling pathways and influencing the phosphorylation and intracellular localization of key signaling molecules. Reduced expression of DNMT1, HDAC6, miR-21, HOTAIR, and H19, while increasing miR-148a/miR-152 expression, suggests that nimbolide regulates the IGF-1/PI3K/Akt signaling pathway through epigenetic modifications. The combined use of nimbolide with metformin and tamoxifen/cisplatin chemotherapeutic agents demonstrated higher efficacy in inhibiting the IGF-1/PI3K/Akt/AR signaling pathways than the use of individual agents [100].

Cisplatin (DDP, cis-diamminedichloroplatinum (II)) resistance in patients with OC poses a serious challenge to successful treatment. Low levels of miR-152 expression in OC correlate with cisplatin resistance, while its restoration increases chemosensitivity by suppressing DNMT1. This mechanism promotes apoptosis and increases the sensitivity of OC cells to cisplatin [101]. Another study [102] demonstrated that the development of cisplatin resistance in OC is associated with the activation of the PI3K/AKT/mTOR signaling pathway through the GAS5/miR-23a-3p/PTEN signaling axis. Studies combining bioinformatics analysis and experimental validation have shown that the expression level of the lncRNA LINC01123 is significantly elevated in OC tissues, and its high expression is associated with poor clinical outcome, as well as chemoresistance, especially to platinum-based drugs and taxanes. Functionally, LINC01123 promotes proliferation, migration, and invasion of OC cells. One of the established mechanisms involves the action of LINC01123 as an endogenous competing RNA (ecRNA) for miR-516b-5p, which leads to increased VEGFA expression, which subsequently stimulates EMT and metastasis [103]. Overexpression of HAND2-AS1 is involved in the restoration of PTEN protein levels and blocks the activation of the PI3K/AKT signaling pathway through the HAND2-AS1/miR-106a/PTEN interaction axis, which increases the sensitivity of cisplatin-resistant OC cells to therapy with this drug [104].

### 4.2. The JAK/STAT Signaling Pathway

The JAK/STAT signaling pathway (Figure 3) also plays a significant role in the development of OC and BC by stimulating EMT, metastasis, and angiogenesis. Furthermore, it is one of the key pathways responsible for chemotherapy resistance. This pathway is initiated by the binding of a ligand (e.g., a cytokine, growth factor, or hormone) to a specific cell surface receptor. Ligand binding leads to receptor dimerization and the activation of associated Janus family tyrosine kinases (JAKs). This leads to the phosphorylation and dimerization of kinase-associated STAT proteins (from STAT1 to STAT6, STAT3 and STAT5 are the most studied). These proteins are transported to the nucleus and directly influence the expression of genes involved in immune regulation, the cell cycle, and therapy resistance. Pathological processes often arise due to mutations in STAT3/5 proteins, but disturbances in the activity of negative regulators of the JAK/STAT signaling pathway are also possible, such as suppressors of cytokine signals (SOCSs), protein inhibitors of activated STATs (PIAS), and protein tyrosine phosphatases (PTPs) [105,106,107].

The acquisition of hypoxia tolerance in BC cells was often accompanied by the activation of the STAT3 transcription factor and persistent overexpression of Snail, a key downstream effector of STAT3. Maintenance and stabilization of the hypoxia-tolerant phenotype are mediated by miR-181a-2, which affects the STAT3/Snail signaling pathway in resistant cells. Inactivation of DNMTs increased cell sensitivity to hypoxia and partially reversed the hypoxia-resistant phenotype of BC, which was accompanied by the activation of the pro-apoptotic p53 signaling pathway, although no significant change in DNA methylase activity was detected in resistant cells. Thus, acquired hypoxia tolerance in BC cells is mediated, at least in part, by the activation of the miR-181a-2/STAT3/Snail signaling pathway, and the use of demethylating agents may represent a promising therapeutic approach to target hypoxia-resistant cancer cell populations [108].

In BC, ADAMTS9-AS1 silencing enhanced cancer cell proliferation and invasion, increased Ki67, PCNA, MMP-9, and MMP-2 levels, and activated the JAK/STAT signaling pathway. Meanwhile, ADAMTS9-AS1 overexpression resulted in miR-301b-3p binding and TGFBR2 activation, inhibiting BC progression through the TGFBR2/JAK/STAT signaling pathway [109].

### 4.3. The Wnt/β-Catenin Signaling Pathway

The Wnt/β-catenin signaling pathway (Figure 3) is activated by Wnt ligand binding to a member of the FZD (frizzled class receptor) family, which leads to the recruitment of LRP5/6 (LDL receptor-related protein 5/6) [110]. Next, the complex, which includes β-catenin, is attracted to the receptor, and β-catenin is released into the cytoplasm, enters the nucleus, and binds to a TCF family protein, which induces the transcription of genes responsible for cell cycle regulation. In the absence of a ligand (e.g., a Wnt signal), β-catenin binds to a destruction complex consisting of AXIN, APC, and GSK3β proteins, which leads to its phosphorylation, ubiquitination, and proteasomal degradation. In the case of OC and BC, pathological processes at this level can be caused by a decrease in the concentration of FZD and LRP dimerization inhibitors (DKK1, SFRP2, CCNG2, DACT1), an increase in the activity of the inhibitory complex (FLIP1L), an increase in the number of ligands for FZD (Wnt) and LRP kinases (CCNY and CDK14), and the release of β-catenin due to the destabilization of the inhibitory complex (TNKS1 and RAB14). Cadherin proteins associated with catenins also play an equally important role. In BC, these are Snail, SLUG, ZEB1, and SIP1, which suppress the expression of E-cadherin, a negative regulator of β-catenin, provoking EMT and metastasis [110,111].

In [112], the effect of miR-205-5p expression changes on LRP6 and the genes of the Wnt/β-catenin signaling pathway expression levels was shown. An increase in the level of miR-205-5p led to a decrease in growth and cell migration and the induction of apoptosis, as well as a decrease in the expression of LRP6, which caused a decrease in the expression of the target genes of the Wnt/β-catenin pathway (c-Myc, CCND1, and PPARδ), and also had a regulatory effect on the expression of lncRNAs MALAT1, NEAT1, SNHG5, and SNHG16. lncRNAs such as MIR4435-2HG, MIR100HG, USP30-AS1, TUG1, BANCR, and others are also involved in the progression of BC through the activation of the Wnt/β-catenin signaling pathway [113,114]. MIR4435-2HG enhances the proliferation, migration, invasion, and EMT of BC cells in vitro, while promoting angiogenesis, tumor growth, and metastasis in vivo [113]. It acts as an endogenous competing RNA to inhibit degradation of the target gene UBE2N by binding miR-205-5p, which promotes BC progression due to the activation of the Wnt/β-catenin signaling pathway. Interaction prediction and expression correlation analysis revealed that MIR100HG could function as a molecular sponge for mir-224-5p, thereby attenuating its suppressive effect on EYA4, which led to the modulation of the Wnt signaling pathway in high-grade serous OC [115]. In one study, lncRNA USP30-AS1 was characterized as a critical factor in promoting tumor stem cell development, chemoresistance, and metastasis in BC cells through the USP30-AS1/miR-3646/FZD7/Wnt axis, suggesting it as a potential therapeutic target [116]. In another study, BANCR activated the HMGA1/Wnt/β-catenin pathway by inhibiting miR-34a-5p, which contributed to the development of therapeutic resistance in BC. In vivo experiments showed that BANCR inhibition slowed tumor progression and reversed trastuzumab resistance [117].

High-throughput mRNA sequencing, bioinformatics analysis, and pharmacological studies have shown that aberrant expression of miR-29c-3p modulates tumorigenesis in OC cells, including EMT, proliferation, migration, and invasion. This modulation occurs through the regulation of the β-catenin signaling pathway by directly targeting the 3′-UTR of DNMT3A, TET1, and HBP1 (HMG-box transcription factor) mRNAs and suppressing their translation [65]. Furthermore, miR-152-3p counteracts EMT by downregulating the expression of metastasis-promoting transcription factors such as ZEB1/2 and SNAI1, limiting tumor invasion and dissemination [118]. In line with these results, researchers found an inverse correlation between DNMT1 mRNA levels and miR-148a and miR-152 expression during BC progression [90]. In the context of BC chemotherapy, miR-152-3p may enhance drug sensitivity by targeting anti-apoptotic proteins (e.g., BCL-2) or drug resistance mediators (e.g., ABCB1) [118]. These data suggest a dual role for miR-152-3p as a promising therapeutic target and prognostic biomarker in BC [118]. Overexpression of miR-152 induces E-cadherin gene CDH1 expression in BC cell lines, as evidenced by increased mRNA and protein levels [119]. Induction of miR-152 expression increases the sensitivity of BC cells to paclitaxel, as evidenced by an increase in the rate of apoptosis and suppression of metastasis and invasion [120,121]. It is worth emphasizing that a decrease in β-catenin expression is accompanied by an increase in miR-152 levels, which further limits cancer cell proliferation in a PKM2-dependent manner [122]. Overexpression of various lncRNAs and circular RNAs that inhibit miR-152 has been observed in many types of cancer. For example, increased CCAT1 expression in epithelial OC mediates its effects through the CCAT1/miR-152/ADAM17 and CCAT1/miR-152/WNT1 axes where CCAT1 sponges miR-152 and further increases the expression of ADAM17 and WNT1, which are involved in the Wnt/β-catenin signaling pathway [123].

### 4.4. The MAPK Signaling Pathway

Both in OC and BC, the MAPK cascade plays an important role (Figure 3). It begins with the activation of the tyrosine kinase receptor, which binds growth factors, leading to the formation of the Ras-GTP (guanosine triphosphate) complex, which acts as a “molecular switch.” This is followed by the sequential activation of Raf, MEK, and ERK factors, which induce the expression of genes associated with cell proliferation, differentiation, migration, senescence, and apoptosis [124]. The polypeptide growth factor EGF activates the membrane tyrosine kinase HER2 by binding to the EGFR receptor on the cytoplasmic membrane, promoting the phosphorylation of MEK and the diphosphorylation of Erk1/2, thereby activating the ERK signaling pathway [125]. In HER2-positive BC, miR-637 can reduce HER2 expression, inhibit MEK phosphorylation and the Erk signaling pathway, and ultimately promote cell apoptosis and inhibit proliferation and differentiation [125].

LncRNAs may be one of the factors modulating the activity of the MAPK/MEK/ERK signaling pathway. LINC01287 is overexpressed in BC, and its regulatory effect may be associated with the LINC01287/miR-98/IGF1R/MEK/ERK signaling pathway. Moreover, knockdown of LINC01287 and overexpression of miR-98 significantly slowed BC progression. Knockdown of LINC01287 also reduced IGF1R levels and phosphorylation of MEK1/2 and ERK1/2 [126]. In [127], high expression of lncRNA PRNCR1 and low expression of miR-377 were observed in BC patients, and patients with the highest PRNCR1 expression had an unfavorable prognosis. Suppression of PRNCR1 expression or increased expression of miR-377 led to the suppression of BC cell proliferation, cell cycle arrest, and apoptosis induction. PRNCR1 regulated CCND2 expression by competitively binding to miR-377. CCND2 activated the MEK/MAPK pathway, and after treatment with Mirdametinib, the MEK/MAPK pathway was inhibited, which slowed BC growth [127].

Other ncRNAs affecting the MEK/MAPK signaling pathway have been identified in OC. The expression of LINC00852 was significantly increased in OC tissues and cell lines, while miR-140-3p was significantly reduced. Knockdown of LINC00852 inhibited the viability, proliferation, and invasion of OC cells and promoted their apoptosis [128]. miR-140-3p interacted with AGTR1 (angiotensin 1 receptor) mRNA and negatively regulated its protein levels in OC tissues, which contributed to the blockade of the MEK/ERK/STAT3 signaling pathway through the LINC00852/miR-140-3p/AGTR1/MEK/ERK/STAT3 axis.

LncRNA LNC00115 contributed to cisplatin resistance, invasion, and the migration of OC cells. LNC00115 was shown to directly target miR-7, whose expression was reduced in OC tissues and cisplatin-resistant OC cell lines. miR-7 increased cisplatin sensitivity, blocked OC cell invasion and migration, and directly targeted the ERK mRNA. ERK expression was increased in cisplatin-resistant cell lines and OC tissues. This study elucidates the mechanism by which the LNC00115/miR-7/ERK axis contributes to cisplatin resistance and identifies a new clinical strategy for combating cisplatin resistance in OC [129].

Epigenetic studies showed that downregulation of miR-193a-3p expression by methylation resulted in a gradual decrease in miR-193a-3p expression from low-grade to high-grade OC cells. miR-193a-3p modulated the expression of GRB7, ERBB4, SOS2, and KRAS in the MAPK/ERK signaling pathway, enhancing the oncogenic properties of OC cells in vitro and in vivo. These data suggest that epigenetic downregulation of miR-193a-3p by DNA hypermethylation is a dynamic process in OC progression, and miR-193a-3p can be used as a promising target for replacement therapy in OC [130].

### 4.5. The NF-κB Signaling Pathway

The NF-κB signaling pathway is closely linked to PI3K and is activated by TNFα, IL-1, and other cytokines. Under the influence of the IKK kinase complex (consisting of IKKα, IKKβ, and the regulatory subunit NEMO/IKKγ), after receiving a signal from the receptor, the inhibitory subunit IkB is released from the dimer of NF-κB family proteins. As a result, the free p50/p65 complex is transported into the nucleus and acts as a transcription factor for genes responsible for blocking apoptosis. This, combined with the PI3K/AKT signaling cascade, can lead to tumor formation. In BC, the NF-κB pathway is often associated with estrogen receptors [131,132]. Another proposed mechanism involves the PKM2/NF-κB/miR-152 pathway, where the miR-152 feedback loop is disrupted due to the hypermethylation of its promoter. In non-cancerous cells, EGR1 production is triggered by the interaction between PKM2 and the NF-κB p65 subunit, which subsequently regulates miR-152 expression. In cancer cells, this pathway is impaired, leading to increased cell proliferation and angiogenesis [133].

The overexpression of many lncRNAs and miRNAs, is involved in modulating the activity of the NF-κB signaling pathway in both BC and OC [134,135,136,137]. LncRNA LINC-PINT exerts its antitumor role in BC through the miR-576-5p/MEIS2/PPP3CC/NF-κB axis [134]. Overexpression of LINC-PINT suppresses proliferation and migration of BC cells. LINC-PINT, by interacting with miR-576-5p, increases the expression of its target gene MEIS2, which positively regulates the catalytic subunit of protein phosphatase gamma 3 (PPP3CC) by inactivating the nuclear factor-κB (NF-κB) pathway [134]. LncRNA CASC9 enhances BC progression by downregulating miR-590-3p and upregulating SIX1 expression during NF-κB signaling pathway activation. These findings suggest that the CASC9/miR-590-3p/SIX1/NF-κB axis is involved in BC progression, providing insight into the role of CASC9 in its development [138]. The authors of [139] demonstrated that constitutive activation of the PCDHB17P/miR-145-3p/MELK/NF-κB feedback loop promotes BC angiogenesis and metastasis, suggesting that this lncRNA may be a promising prognostic biomarker and therapeutic target. Overexpression of the lncRNA LOXL1-AS1 promotes BC invasion and metastasis by blocking the expression and activity of its target miR-708-5p, which leads to increased nuclear factor κB (NF-κB) activity [137]. The authors of [140] suggest that miR-221 promotes tumorigenesis in BC by regulating stemness, at least in part, by controlling DNMT3b expression, which in turn controls the expression of various stem cell genes, such as Nanog and Oct 3/4, by affecting their promoter methylation.

Scoparone (SCO), a compound found in the stems and leaves of Artemisia capillaris, is a promising pharmacological agent for the antitumor therapy of BC. The results in [141] showed that SCO exerts a time- and dose-dependent inhibitory effect on BC cell viability and that increased expression of the lncRNA SNHG12 in BC cells is suppressed by SCO. SNHG12, which is predominantly expressed in the cytoplasm, acted as a ceRNA, binding miR-140-3p and inhibiting its expression. The transcriptional activity and translational level of TRAF2, a downstream target of miR-140-3p, were reduced following SCO-mediated suppression of SNHG12 expression. Reduced TRAF2 activity resulted in inhibition of the NF-κB signaling pathway, decreased viability and migration of BC cells, and apoptosis stimulation [141].

With regard to OC, several studies have been conducted to identify lncRNAs and miRNAs that trigger the activation of the NF-κB signaling pathway. LncRNA MAFG-AS1, by binding miR-339-5p, promotes the upregulation of NFKB1 and IGF1 by recruiting NFKB1. Thus, overexpression of MAFG-AS1 accelerates the EMT, invasion, and migration of OC cells, which can be prevented through the suppression of IGF1 or NFKB1 [136]. The level of HOTTIP was significantly increased in cisplatin-resistant cells, and its suppression abolished cisplatin resistance in these cells [142]. HOTTIP was found to bind miR-205, so that cells with suppressed HOTTIP levels had higher levels of miR-205. HOTTIP silencing also resulted in decreased NF-κB activation, clonogenic potential, and reduced expression of the stem cell markers SOX2, ZEB2, OCT4, and NANOG, an effect that could be attenuated by miR-205. Finally, the authors identified ZEB2 as a miR-205 target gene, establishing the HOTTIP/miR-205/ZEB2 axis as a novel functional axis involved in determining cisplatin resistance in OC cells [142]. LncRNA PRLB has been identified as a factor that enhances drug resistance in OC cells [143]. PRLB silencing has been shown to enhance OC cell sensitivity to paclitaxel, at least in part, by inhibiting the activation of the RSF1/NF-κB signaling pathway through targeting miR-150-5p.

LINC01123 contributes to chemoresistance in OC, particularly to platinum-based drugs and taxanes. Although the exact mechanisms are still under investigation, a potential link to EMT regulation, autophagy modulation, and altered glycolysis has been suggested. For example, studies on Berberine, an alkaloid with anticancer properties, have shown that it can inhibit glycolysis and autophagy in OC cells, potentially affecting the LINC01123/NF-κB/MAPK10 signaling pathway [135]. These data implicate LINC01123 as a factor contributing to OC aggressiveness and treatment failure.

### 4.6. The Nrf2 Signaling Pathway

Nrf2 (Nuclear factor erythroid 2-related factor 2) and ARE (Antioxidant Response Element) are jointly responsible for protecting cells from oxidative stress, inflammation, and toxins, controlling the expression of over 500 genes involved in antioxidant defense, detoxification, and cellular homeostasis. Normally, Nrf2 binds to KEAP1 (kelch-like ECH associated protein 1), leading to its proteasomal degradation. However, cellular stress, KEAP1 mutations, or increased miR-181d levels can lead to hyperactivation of Nrf2 and, in the case of OC and BC, to chemotherapy resistance [144,145].

### 4.7. The HIF-1α Signaling Pathway

The signaling cascade associated with the HIF-1α protein (HIF1A, hypoxia inducible factor 1 subunit alpha) like NF-κB is inextricably linked to the PI3K/AKT signaling cascade and is activated by the mTOR complex. It downregulates many proteins that inhibit carcinogenesis, such as p53 and Bax (activating apoptosis), and induces proteins that promote oncogenesis, including Bcl-2 (preventing apoptosis), LDH (lactate dehydrogenase, involved in anaerobic glycolysis), Nptx2 (responsible for the production of the inflammatory factor IL-6), p38 and Vegf1 (stimulating proliferation), and AEG1 (inducing MMPs 2 and 9, which facilitate metastasis). The activation of HIF-1α is promoted by ncRNAs. For example, the previously discussed JAK/STAT cascade is responsible for stimulating the lncRNA DSCR8, which in turn binds to miR-98-5p and prevents the inhibition of its target gene, HIF1A. For some types of BC and OC, HIF-1α is a promising target for preventing invasion and metastasis [146,147].

In triple-negative BC, a positive correlation was observed between lncRNA MT1JP and miR-138, but a negative correlation with HIF-1α. Moreover, overexpression of miR-138 resulted in a decrease in HIF-1α levels, but did not affect MT1JP expression. With high expression levels of lncRNA MT1JP and miR-138, a decrease in the proliferation rate of triple-negative BC cells was observed [148]. MT1JP inhibits triple-negative BC by regulating the miR-138/HIF-1α axis and may serve as a biomarker or target for the treatment of triple-negative BC [148]. A study [149] showed that lncRNA VCAN-AS1 was overexpressed in BC tissues and cell lines, while the expression level of miR-106a-5p was reduced and negatively correlated with the expression level of VCAN-AS1. High levels of VCAN-AS1 accelerated proliferation, migration, invasion, and EMT and suppressed apoptosis, and were also correlated with poor overall survival in BC patients. Using bioinformatics analysis, the authors found that VCAN-AS1 functioned as a ceRNA for miR-106a-5p, which targets the 3′-UTR of the STAT3 mRNA, thereby suppressing the STAT3/HIF-1α pathway, while activation of the STAT3 pathway resulted in the suppression of the antitumor effects mediated by miR-106a-5p. This study opens new avenues for BC therapy [149]. In [150], the authors showed that the expression levels of MALAT1 were significantly increased under hypoxic conditions and were regulated by HIF-1α and HIF-2α as transcription factors. Furthermore, miRNA expression levels and RNA immunoprecipitation using anti-AGO2 antibodies revealed that MALAT1 acts like a sponge for miR-3064-5p, which targeted HIF-1α and HIF-2α mRNA. Finally, functional assays revealed that MALAT1 can promote the migration and proliferation of BC cells in culture. These data suggest that MALAT1 may be a candidate for therapeutic intervention to slow the progression of BC [150].

In OC, high expression of lncRNA DSCR8 increased the levels of STAT3 and HIF-1α, while attenuating the effect of miR-98-5p, which promoted cell proliferation, invasion, and EMT and suppressed apoptosis. Pharmaceutical targeting of STAT3 and HIF-1α significantly altered the expression of DSCR8 and miR-98-5p. This information suggests the presence of a positive feedback loop between the lncRNA DSCR8/miR-98-5p/STAT3/HIF-α axis and OC progression [151]. In [152], the authors demonstrated that LINC00662 acts as a ceRNA and can modulate HIF-1α expression through direct binding to miR-375, which targets HIF-1α mRNA. These data suggest that LINC00662 may be investigated as a predictive biomarker for OC in anti- HIF-1α therapy [152].

### 4.8. The Notch Signaling Pathway

The Notch signaling cascade (Figure 3) plays a key role in angiogenesis during the progression of OC and BC. It involves information exchange between two cells in the system: the “signaler” and “receiver”. The pathway is activated when transmembrane Notch receptors on one cell interact with ligands (e.g., from the Delta-Serrate-LAG-2 family) on an adjacent cell. This interaction leads to proteolytic cleavage of the Notch receptor, releasing its intracellular domain (NICD), which then translocates to the nucleus. In the nucleus, NICD forms a complex with the DNA-binding protein CBF1/RBP-Jκ and other cofactors, altering the expression of target genes such as HES1, HEY1, c-Myc, and others responsible for vascular network formation. Notch is closely associated with other oncogenic pathways and is regulated by VEGF (vascular endothelial growth factor) [153,154].

Elevated levels of Jagged-1 (JAG1), a canonical ligand of the Notch signaling pathway, are an unfavorable prognostic factor and have a proinvasive effect in triple-negative BC. JAG1 promotes angiogenesis and is involved in the formation of the microenvironment by stimulating exosome secretion through the MALAT1/miR-140-5p/JAG1/VEGFA axis [155]. In OC tissues and cell lines, the expression of SNHG3 and Notch1 was significantly increased, while that of miR-139-5p was significantly decreased. Inhibition of SNHG3 suppressed the proliferation and migration of cells in the OVCAR3 OC cell line. A luciferase reporter experiment confirmed that miR-139-5p could target SNHG3 and Notch1. Transfection of miR-139-5p inhibitor significantly attenuated the inhibitory effect of SNHG3 knockdown on OVCAR3 cell proliferation and migration. Moreover, SNHG3 inhibition or miR-139-5p mimic abolished the stimulatory effect of Notch1 overexpression on OVCAR3 cell proliferation and migration. Thus, SNHG3 could accelerate OC cell proliferation and migration by regulating miR-139-5p and Notch1 [156].

### 4.9. The Hippo Signaling Pathway

Hippo is a signaling pathway involved in many oncogenic processes, as well as at certain stages of embryogenesis, through the activity of an enzymatic complex consisting of four proteins: MST, SAV1, MOB1, and LATS. It is regulated by both GPCR receptors and integrins through the modulation of RHO-GTPase and by the PI3K signaling pathway. When the Hippo pathway is activated, YAP and TAZ proteins are phosphorylated, preventing their translocation to the cell nucleus. In the nucleus, these proteins typically interact with transcription factors of the TEAD (transcriptional enhancer activation domain) family, activating genes responsible for cell proliferation and growth. YAP1 is one of the most promising biomarkers in the context of OC and BC [157,158].

LncRNA A2M-AS1 accelerates the proliferation, invasion, and colony formation of BC cells and suppresses apoptosis through the miR-146b/MUC19/Hippo axis, which was confirmed in vivo [159]. LINC00673 is another lncRNA associated with poor prognosis in BC, activation of cell proliferation, and suppression of apoptosis. Suppression of LINC00673 inhibits BC cell proliferation by suppressing the cell cycle and triggering apoptosis. Therapy using antisense oligonucleotides (ASOs) targeting LINC00673 significantly suppresses BC cell proliferation in vivo. The transcription factor Yin Yang 1 (YY1) binds to the promoter of the LINC00673 gene and increases its transcription, resulting in decreased miR-515-5p levels, increased MARK4 expression, and subsequent inhibition of the Hippo signaling pathway [160].

In OC, regulation of the Hippo/YAP signaling pathway is less well understood. The study [161] demonstrated that lncRNA ASAP1-IT1 expression was reduced in OC samples and OC cell lines. Overexpression of ASAP1-IT1 inhibited ovarian cell proliferation and induced apoptosis. Bioinformatic analysis predicted that miR-2278, a previously described miRNA with increased expression in OC, may bind to ASAP1-IT1. Furthermore, data showed that the target gene of miR-2278 is LATS2, the expression of which was increased by ASAP1-IT1 in OC cells. By regulating LATS2 expression, ASAP1-IT1 induced a decrease in YAP1 expression in OC cells. Suppression of LATS2 expression attenuates the inhibition of cell proliferation and apoptosis induced by ASAP1-IT1 overexpression in OC cells [161].

### 4.10. The TGF-β Signaling Pathway

The TGF-β signaling pathway begins with the binding of TGF-β to its type II receptor, which then activates the type I receptor. Following activation, the type I receptor is phosphorylated and transmits a signal into the cell through proteins of the SMAD family. This cascade plays a key role in the regulation of cell proliferation, differentiation, apoptosis, embryogenesis, and immune function and is involved in the progression of OC and BC through interactions with the MAPK and PI3K/AKT/mTOR pathways [109,162,163,164].

LINC00536 is overexpressed in BC tissues and cell lines, and its expression correlates with a poor prognosis. High levels of LINC00536 are associated with increased stromal immune cell counts and immune checkpoint gene expression, suggesting a potential role in modulating the tumor microenvironment. LINC00536 bound miR-204-5p, thereby upregulating TGFBR2 expression, which activated the TGF-β signaling pathway and induced EMT. In vitro, LINC00536 promoted proliferation, migration, and EMT, while in vivo, it accelerated tumor growth and lung metastasis through the LINC00536/miR-204-5p/TGFBR2 axis [165]. XIST is involved in the activation of the TGF-β/SMAD signaling pathway in BC. Knockdown of XIST suppressed the proliferation, invasion, migration, and EMT of BC cells and induced cell cycle arrest in the G0/G1 phase through the XIST/miR-455-3p/HOXC4 axis. Further experimental data in vivo showed that downregulation of XIST expression reduced the tumorigenicity of BC cells and decreased the expression of HOXC4 and p-SMAD3 (a protein associated with the TGF-β/SMAD signaling pathway) [166]. Another factor activating the TGF-β signaling pathway is the LINC00052/miR-145-5p/GFBR2 axis stimulating the proliferation and metastasis of BC cells. Therefore, LINC00052 may be an effective potential target for BC theranostics [167].

Metastasis is a major cause of death and chemotherapy failure in patients with BC. The authors of [168] showed that treatment of BC cell lines with baicalin, obtained from the roots of Scutellaria baicalensis, inhibited cells viability, as well as their migration and invasion. Treatment with baicalin significantly reduced the expression of TGF-β, ZEB1, and N-cadherin and increased the expression of E-cadherin at both the mRNA and protein levels. In addition, treatment with baicalin significantly reduced the expression of MALAT1 and increased the expression of miR-200c. Thus, baicalin significantly suppresses the viability, migration, and invasion of BC cells, possibly by regulating the MALAT1/miR-200c/TGF-β signaling pathway [168].

In OC, activation of ERK1/2, Smad2, and Smad4 was found to be associated with the PVT1/miR-148a-3p/AGO1/TGF-β pathway-induced cascades, highlighting its potential as a valuable target for OC anticancer therapy [169]. Upregulation of LINC02323 and downregulation of miR-1343-3p were observed in OC patients. Overexpression of LINC02323 promoted cell growth and decreased LDH activity in an in vitro model by downregulating miR-1343-3p expression and inducing TGF-β1 receptor, which promoted cell growth [170]. TET3 gene expression was downregulated during TGF-β1-induced EMT in SKOV3 and 3AO OC cell lines. Overexpression of TET3 restored the epithelial phenotype after TGF-β1-induced EMT, including changes in the expression patterns of its molecular markers (E-cadherin, vimentin, N-cadherin, Snail) in the migratory and invasive abilities of OC cells. High levels of TET3 led to demethylation in the promoter region of the gene encoding miR-30d, which resulted in the restoration of miR-30d (EMT suppressor) levels. TET3 expression was inversely correlated with the degree of OC differentiation [41].

### 4.11. The P53 Signaling Pathway

The p53 protein, encoded by the TP53 gene (Tumor Protein p53), is considered the main conductor of intracellular processes and signaling pathways. It plays a fundamental role in the prevention of carcinogenesis, as well as in the aging process [171]. The occurrence of mutations in TP53 promotes the survival of cells with damaged DNA, as well as their active proliferation and subsequent malignant transformation. Normally, cellular stress or DNA damage signals lead to the activation of p53 and the transcriptional regulation of p21/CDKN1A, a CDK (cyclin-dependent kinase) inhibitor. p21 modifies the RB protein via phosphorylation. Activation of p16 INK4A controls the phosphorylation of RB by binding to CDK4/6 and inhibiting cyclin D. RB then interacts with E2F, limiting the transcription of genes necessary for the transition from the G1 phase of the cell cycle to the G2 phase, and then to the M phase. Loss of p53 function significantly accelerates the oncogenesis process, creating favorable conditions for the development of BC and OC. Thus, it is potentially possible to create targeted therapy and prognostic markers based on the modulation of p53 protein activity [171,172,173]. LEMD1-AS1 expression was reduced in OC tissues and cell lines. Forced overexpression of LEMD1-AS1 inhibited the proliferation, migration, and invasion of OC cells, as well as the growth of transplanted tumors in nude mice. LEMD1-AS1 can directly interact with miR-183-5p and the p53 protein, and the antitumor role of LEMD1-AS1 in OC progression depended on miR-183-5p-mediated TP53 gene expression [174].

Thus, the interaction of canonical signaling pathways of carcinogenesis and their epigenetic regulators (DNA methylation/demethylation enzymes, miRNAs, and lncRNAs) creates a unique molecular profile that is critical for the selection of prognostic and predictive oncomarkers and targeted anticancer therapy.

## 5. Discussion

Unlike static genetic variants, epigenetic marks are dynamic, context-dependent, and potentially reversible, making them particularly suitable for early risk stratification and preventive intervention. Accordingly, future diagnostic, predictive, and prognostic systems need to go beyond genomics and incorporate epigenetic information to reflect the current molecular state and future disease trajectories.

The analysis revealed that key epigenetic mechanisms in BC and OC form a single multi-level regulatory system, including DNA methylation and demethylation enzymes, miRNAs, lncRNAs, and their target genes. Despite the differences between these tumor types, most of the identified interactions can be grouped into several regulatory circuits.

The first circuit is associated with an imbalance between DNA methylation and demethylation processes. Overexpression of DNMT1, DNMT3A, and DNMT3B leads to hypermethylation of the promoter regions of tumor suppressor genes (BRCA1, PTEN, RASSF1A, CDH1, and others), which promotes proliferation, invasion, and metastasis, as well as the development of drug resistance. Conversely, decreased activity of TET enzymes is accompanied by a decrease in 5hmC levels and disruption of the expression of genes controlling differentiation, apoptosis, and intercellular adhesion, which also contributes to tumor progression.

The second circuit is represented by a feedback loop between miRNAs and methylation/demethylation enzymes. Several miRNAs (miR-152, miR-145, miR-29c, and others) can directly inhibit the expression of DNMTs, reducing aberrant methylation levels and restoring the expression of tumor suppressors (Figure 1). In turn, hypermethylation of miRNA genes leads to a decrease in their expression and the activation of oncogenic programs.

The third circuit is formed by lncRNA-miRNA-mRNA interactions. Both tumor types are characterized by the involvement of lncRNAs as ceRNAs, binding miRNAs and altering the expression of their target genes. The most significant examples are the NEAT1/miR-214-3p/SEMA4D, HOTAIR/miR-214-3p/miR-217/PIK3R3, and GAS5/miR-23a-3p/PTEN axes in OC, as well as the FOXD3-AS1/miR-363/TFF1, MIR497HG/miR-497/miR-195/AKT-related targets, and TTN-AS1/miR-107/ZNRF2 axes in BC. Such interactions regulate proliferation, apoptosis, migration, invasion, and the development of drug resistance.

The fourth circuit involves interactions between lncRNAs and enzymes involved in epigenetic DNA modification. Some lncRNAs can directly regulate the activity of DNMTs or TETs, altering the methylation profile of target genes. These mechanisms include the LINC00518/DNMT/CDX2, H19/SAHH/DNMT3B/BECN1 axes, and several others (Figure 1). Such interactions provide a link between epigenetic changes and the functioning of signaling cascades involved in carcinogenesis.

The final fifth circuit includes key signaling pathways that are the endpoints of most of the epigenetic interactions described in this review. The most frequently implicated cascades are the PI3K/AKT/mTOR, Wnt/β-catenin, and EMT-associated programs, as well as pathways regulating angiogenesis and cell survival. These signaling systems serve as a functional bridge between epigenetic disturbances and the formation of an aggressive tumor phenotype. Thus, despite the significant diversity of individual molecular interactions, most of the identified epigenetic abnormalities in BC and OC are assembled into a limited number of regulatory circuits that integrate DNA methylation and demethylation enzymes, miRNAs, lncRNAs, and key signaling cascades. These data suggest that the axis of “DNMT/TET-lncRNA-miRNA-signaling pathways” can be considered a unified functional system, likely one of the key regulators involved in the formation of tumor phenotype, metastatic potential, and sensitivity to therapy in BC and OC. Further study of the key components of this system may facilitate the development of new diagnostic, prognostic, and therapeutic approaches for patients with BC and OC.

Potential therapeutic strategies include the correction of methylase and demethylase activity, including the restoration of TET1/2/3 expression suppressed by oncogenic miRNAs (miR-9-5p, miR-17-5p, etc.), which may promote the demethylation of tumor suppressor promoters such as BRCA1/2, PTEN, and TP53. Similar approaches are being explored in the context of CRISPR-Cas9 and CRISPRa technologies aimed at regulating the transcriptional activity of the corresponding genetic loci [175].

An additional avenue is the inhibition of oncogenic miRNAs using CRISPR-Cas13 systems or antisense oligonucleotides, which prevents the suppression of TET family gene expression. Meanwhile, the activation of suppressor lncRNAs of the SNHG family (SNHG1, SNHG6, SNHG7) using modified CRISPR systems, viral vectors, or nanoparticles can be considered a way to alter the epigenetic profile of tumor cells [176].

Therapy targeting DNMT enzymes is also of considerable interest. The interaction of SNHG lncRNAs with DNMT1 and DNMT3B justifies the use of DNA methyltransferase inhibitors, such as 5-azacytidine and decitabine, in combination with approaches that affect the expression of regulatory ncRNAs [177,178]. Similarly, miR-9-5p, miR-125b-5p, and miR-17-5p can be considered potential targets for antisense therapy [179,180]. Furthermore, epigenetic vaccines [181] and RNA-editing therapies [182], aimed at correcting dysregulated ncRNAs without direct intervention in the genome, remain promising approaches, although still in the early stages of development.

Taken together, the presented data highlight the importance of a comprehensive study of interactions between non-coding RNAs and epigenetic enzymes as a basis for developing personalized strategies for the diagnosis and treatment of oncological diseases.

## 6. Conclusions

Emerging evidence underscores that epigenetic abnormalities in BC and OC are orchestrated not by isolated events but by a highly integrated multi-level regulatory system. This review conceptualizes these interactions into five core regulatory circuits where DNMTs and TET demethylases engage in reciprocal feedback loops with oncogenic and suppressor ncRNAs (miRNAs and lncRNAs). This unified “DNMT/TET–lncRNA–miRNA” axis effectively governs key downstream oncogenic cascades, including the PI3K/Akt/mTOR and Wnt/β-catenin pathways, driving tumor progression and drug resistance. Given the inherent plasticity and reversibility of epigenetic marks compared to static genetic mutations, this axis represents a premier target for precision medicine. Future clinical breakthroughs rely on moving beyond genomics to integrate dynamic epigenetic profiling into diagnostic frameworks. Furthermore, exploiting these vulnerabilities via advanced combinatorial strategies—such as by integrating traditional hypomethylating agents with RNA-targeted therapies, ASOs, and CRISPR-mediated epigenome/RNA editing systems (CRISPR-Cas9/a/13)—is very promising for overcoming therapeutic resistance and revolutionizing personalized oncology.

## Data Availability

No new data were created or analyzed in this study. Data sharing is not applicable to this article.

## Abbreviations

The following abbreviations are used in this manuscript:

BC	breast cancer
OC	ovarian cancer
DNMTs	DNA methyltransferases
TET	Ten-Eleven translocation enzymes
5hmC	5-hydroxymethylcytosine
AP site	apurinic–apyrimidinic site
BER	base excision repair
ncRNA	non-coding RNA
lncRNA	long non-coding RNA
miRNA	microRNA
mRNA	messenger RNA
EMT	epithelial–mesenchymal transition
ASOs	antisense oligonucleotides
3′-UTR	3′-untranslated region
ceRNA	competing endogenous RNA
5mC	5-methylcytosine
5hmC	5-hydroxymethyl cytosine
5fC	5-formylcytosine
5caC	5-carboxycytosine
TDG	thymine DNA glycosylase
UNG	uracil DNA glycosylase
SMUG1	single strand selective monofunctional uracil DNA glycosylase
MBD4	methyl CpG binding domain 4, DNA glycosylase
PTEN	phosphatase and tensin homolog
(ER+) BC	estrogen receptor-positive breast cancer
FZD	frizzled class receptor
LRP5/6	LDL receptor related protein 5/6
NF-κB	nuclear factor κB
HIF-1α	HIF1A, hypoxia inducible factor 1 subunit alpha
